# Phylogeographic structure of the dunes sagebrush lizard, an endemic habitat specialist

**DOI:** 10.1371/journal.pone.0238194

**Published:** 2020-09-16

**Authors:** Lauren M. Chan, Charles W. Painter, Michael T. Hill, Toby J. Hibbitts, Daniel J. Leavitt, Wade A. Ryberg, Danielle Walkup, Lee A. Fitzgerald

**Affiliations:** 1 Department of Biology, Pacific University, Forest Grove, Oregon, United States of America; 2 Endangered Species Program, New Mexico Department of Game and Fish, Santa Fe, New Mexico, United States of America; 3 Albuquerque, New Mexico, United States of America; 4 Department of Ecology and Conservation Biology, Biodiversity Research and Teaching Collections, Texas A&M University, College Station, Texas, United States of America; 5 Natural Resources Institute, Texas A&M University, College Station, Texas, United States of America; 6 Natural Resources Program, Naval Facilities Engineering Command South West, San Diego, California, United States of America; 7 Natural Resources Institute, Texas A&M University, College Station, Texas, United States of America; 8 EEB PhD Program, Texas A&M University, College Station, Texas, United States of America; National Cheng Kung University, TAIWAN

## Abstract

Phylogeographic divergence and population genetic diversity within species reflect the impacts of habitat connectivity, demographics, and landscape level processes in both the recent and distant past. Characterizing patterns of differentiation across the geographic range of a species provides insight on the roles of organismal and environmental traits in evolutionary divergence and future population persistence. This is particularly true of habitat specialists where habitat availability and resource dependence may result in pronounced genetic structure as well as increased population vulnerability. We use DNA sequence data as well as microsatellite genotypes to estimate range-wide phylogeographic divergence, historical population connectivity, and historical demographics in an endemic habitat specialist, the dunes sagebrush lizard (*Sceloporus arenicolus*). This species is found exclusively in dune blowouts and patches of open sand within the shinnery oak-sand dune ecosystem of southeastern New Mexico and adjacent Texas. We find evidence of phylogeographic structure consistent with breaks and constrictions in suitable habitat at the range-wide scale. In addition, we find support for a dynamic and variable evolutionary history across the range of *S*. *arenicolus*. Populations in the Monahans Sandhills have deeply divergent lineages consistent with long-term demographic stability. In contrast, populations in the Mescalero Sands are not highly differentiated, though we do find evidence of demographic expansion in some regions and relative demographic stability in others. Phylogeographic history and population genetic differentiation in this species has been shaped by the configuration of habitat patches within a geologically complex and historically dynamic landscape. Our findings identify regions as genetically distinctive conservation units as well as underscore the genetic and demographic history of different lineages of *S*. *arenicolus*.

## Introduction

Patterns of population genetic diversity within species are shaped by both evolutionary and contemporary history [1]. Though anthropogenic changes to landscapes alter patterns of connectivity that can result in the divergence or coalescence of populations, these processes take place on a background of evolutionary history determined by chance, species’ life history, and also geologic and climatic changes. Characterizing this evolutionary history and identifying the role that organismal traits, evolutionary processes, and ecological conditions have on patterns of phylogeographic divergence adds to our understanding of evolution and is also fundamental to conserving evolutionary potential in the face of anthropogenic disturbance and climate change [2].

The phylogeographic history of species can reflect the roles that habitat connectivity, gene flow, and population stability have played in a species’ evolutionary persistence. Some species may be characterized by deeply divergent lineages, suggesting a history of limited dispersal and low connectivity among sites (e.g., [3–5]), especially in ecosystems with steep environmental gradients and discontinuous habitat [6]. Plant and animal taxa in naturally fragmented landscapes can exhibit strong patterns of genetic population structure with selection favoring limited dispersal. Phylogeographic analyses of *Stenopelmatus* species (Jerusalem crickets) in southwestern North America, for example, revealed limited dispersal among populations, and identified a recent response to anthropogenic change [7]. A meta-analysis of genetic diversity among 21 species of terrestrial animals identified hotspots of genetic diversity that may also be regions with high levels of trait divergence due to natural selection [6]. Alternatively, populations may be only weakly divergent across a species’ range indicating high connectivity (e.g. [8,9]) even in the face of strong local dynamics (e.g. [10]). Identifying evolutionary scenarios and processes that have resulted in particular phylogeographic patterns can help us disentangle processes that underlie population genetic divergence from those that maintain genetic diversity. Understanding the drivers of population genetic structure across the range of a species can also help us predict the response to loss of habitat and the overall vulnerability of species to anthropogenic landscape change.

Ecological specialists can have greater population genetic and phylogeographic structure than generalists because individuals and populations may be restricted to spatially isolated patches of suitable habitat [11–13]. Ecological specialists may have narrow physiological tolerances, specific habitat requirements, and be locally abundant but rare at regional scales [14]. Habitat specialists use specific landscape features and vegetation associations within their range, and often possess eco-morphological and behavioral adaptations [15,16]. Traits that make habitat specialists well-suited for a narrow habitat niche also tend to make them relatively poor dispersers [17]. Low tolerance for unsuitable landscapes is expected to restrict movements among isolated patches of preferred habitat. Ecological studies focusing on the demography and distribution of habitat specialists have found they are sensitive to landscape fragmentation [18,19].

Local processes are often linked to patterns observed across long-term, evolutionary time scales and at broader spatial scales (see reviews by [1,20]). Thus, in specialists with strict habitat specificity and limited dispersal among populations, we might expect phylogeographic structure to reflect historical patterns of divergence and low population connectivity overall (e.g., [11]). Alternatively, habitat specialists may have well-connected populations throughout their range over longer time-scales, indicating a strong role for dispersal and migration that counters the fine-scale divergence of isolated local populations [10]. Characterizing evolutionary patterns of divergence and historical demographics in habitat specialists can help us predict the role that short and long-term dynamics play in shaping population genetic structure. In addition, describing spatial patterns of diversity and identifying independent evolutionary units, historical barriers to gene flow, bottlenecks and founder events, and regions of high connectivity allows the effects of contemporary pressures to be disentangled from historical drivers and also provides important information for the future management and conservation of species.

**Fig 1:**
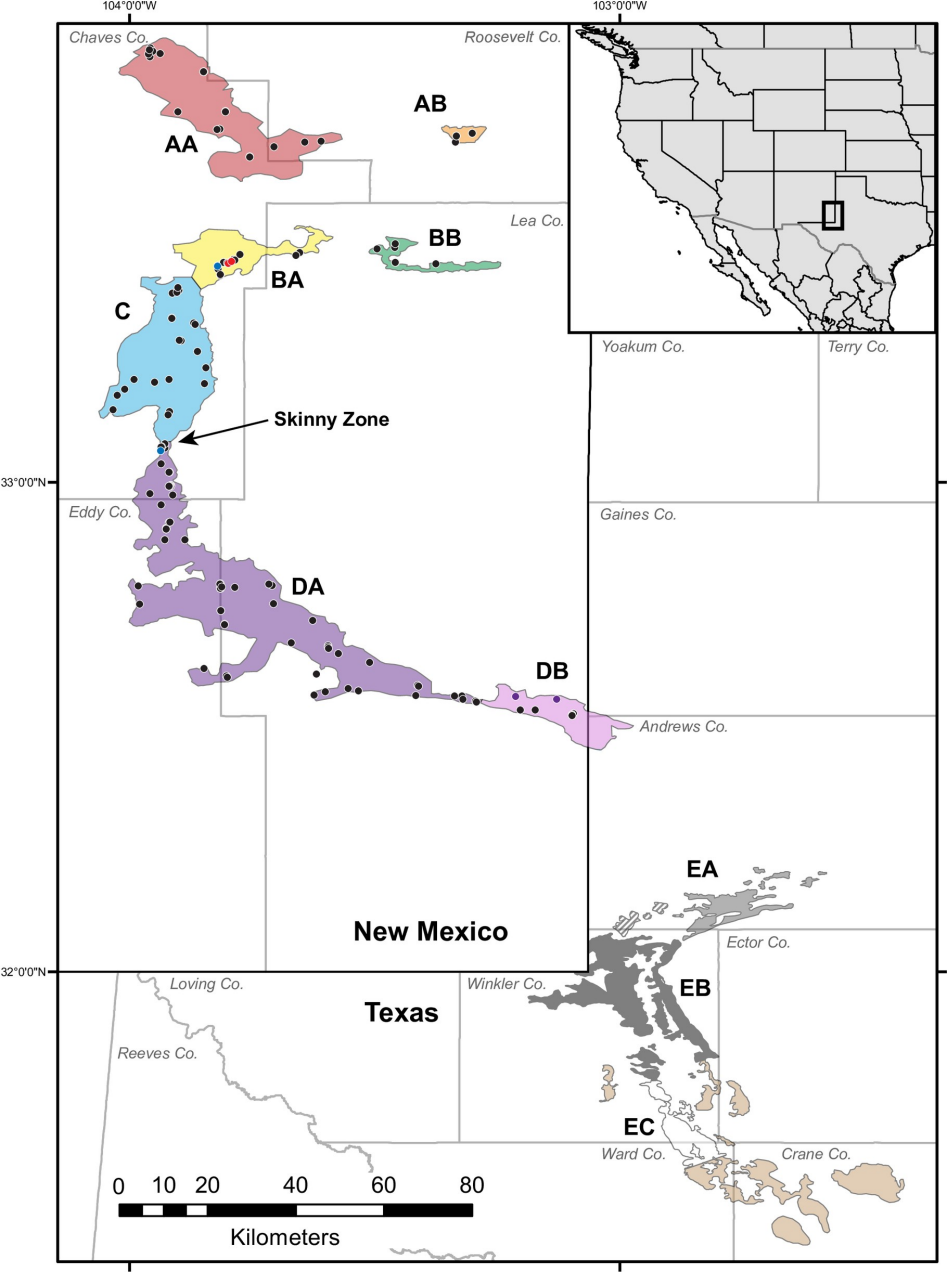
Suitable shinnery oak-sand dune habitat for *S*. *arenicolus* (from [22]) and sampling localities for this study. Black and gray lines indicate state and county boundaries, respectively. Shaded areas delineate potentially suitable habitat for *S*. *arenicolus* with colored portions of the landscape corresponding to the phylogroups and geographic regions referred to in the text. Striped areas have not been surveyed, but contain suitable habitat. Brown indicates areas with potentially suitable habitat where *S*. *arenicolus* has not been found. One locality exists in Crane County from 1970, but the species has not been found there since then. Collection localities for samples of *S*. *arenicolus* from New Mexico included in this study are indicated with points. Specific localities are not shown for Texas due to legal confidentiality agreements with landowners. Collection localities indicated by colored (non-black) points are samples with mitochondrial haplotypes more closely related to haplotypes from other regions.

The dunes sagebrush lizard, *Sceloporus arenicolus*, is endemic to the Mescalero and Monahans Sandhills ecosystem of southeastern New Mexico and adjacent Texas [21,22] (Fig 1). This species is part of the *Sceloporus graciosus* clade [5]. In contrast to other members of this group which tend to be geographically widespread generalists, *S*. *arenicolus* is a habitat specialist. Within this ecosystem, it only uses shinnery-oak sand dune formations with interconnected dune blowouts (sandy depressions created by wind) and in some cases shinnery hummocks in dunes with steep slopes [22–24]. In the Mescalero-Monahans Sandhills Ecosystem, dune blowouts are emergent landforms that are maintained by the interactions among wind, moving sand, and the shinnery oak (*Quercus havardii*) which stabilizes the dunes [25]. Individual *S*. *arenicolus* lizards demonstrate a nested hierarchy of habitat selection [23], selecting for thermally suitable microhabitats and having preference for relatively large dune blowouts. A sand-diving species, they do not occur in areas with relatively fine sand [23,26]. At the highest level of habitat selection, they are endemic to the narrowly distributed Mescalero-Monahans Sandhills [21].

Specialists can reach high population densities in their preferred habitat, and can outcompete generalists in the same area even in some degraded habitats [27,28]. This is true too for *S*. *arenicolus*, where populations of this ecological specialist thrive where the configuration of key landscape features supports larger groups of interacting individuals, defined as neighborhoods (*sensu* [29,30]). Intensive surveys, like those by Walkup et al. [31], have determined habitat occupancy throughout the range, and numerous, detailed demographic studies (e.g. [18,24,25,32,33]) have helped us to understand how individuals and population dynamics are tightly linked to the configuration of landforms in this heterogeneous landscape. Diffusion dispersal throughout interconnected areas of suitable habitat appear key to maintaining populations in contiguous habitat over the long term [30]. The quantity of habitat is positively correlated with the quality of habitat [34], and the occurrence of *S*. *arenicolus* is associated with relatively large core areas of shinnery oak dunes.

Since at least the 1930s, anthropogenic disturbances from herbicide spraying, oil and gas mining, and more recently, sand-mining, have resulted in fragmentation and degradation of the shinnery oak dunes. Long-term monitoring and extensive fieldwork also demonstrate that fragmentation of the shinnery oak dunelands leads directly to population collapse because quality of habitat tends to degrade in response to fragmentation, and dispersal is disrupted [18,19].

To adequately inform conservation and management actions, it is necessary to understand the evolutionary history of this species at both broad and fine-scales throughout the range. Previous genetic work confirmed that at broad spatial scales, *S*. *arenicolus* is comprised of at least three distinct genetic groups [35]. However, it is unclear where these genetic breaks occur geographically and whether they coincide with putative natural or man-made barriers to movement. The purpose of this study is to characterize the evolutionary history of the dunes sagebrush lizard using more complete geographic and genetic sampling. To identify evolutionary distinct geographic lineages and to reconstruct the population history of these lineages, we evaluate mitochondrial and nuclear sequence data as well as multilocus microsatellite genotypes. Sampling for individuals occurred evenly throughout the entire known range of this endemic and threatened lizard.

## Materials and methods

### Sampling

This study was carried out in strict accordance with the recommendations in the Guide for the Care and Use of Laboratory Animals of the National Institutes of Health. The protocols were approved by the Institutional Animal Care and Use Committees at Duke University (Protocol: A065-12-03), the Claremont Colleges (protocol number not assigned), Texas A&M University (Protocols: AUP 2008–95, AUP-2011-130, AUP 2012–105), and Pacific University (Protocol: R-0026). Animals were captured and released, or euthanized with injection by MS222. Field research was approved by New Mexico Department of Game and Fish (#3588 to LMC, #1755 to LAF, #3353 to MTH, #3409 to DJL) and Texas Parks and Wildlife Department (SPR 0397–867 to LAF, SPR 0506–662 to TJH).

We surveyed for *Sceloporus arenicolus* throughout their range (Fig 1). Liver or muscle tissue was collected from vouchered specimens deposited in the Biodiversity Research and Teaching Collections of Texas A&M University (symbolic code: TCWC), or Museum of Southwestern Biology (MSB). Additionally, toe and/or tail tips were collected non-destructively from animals caught in the field that were subsequently released. All tissue samples were stored in 95% EtOH. Whole genomic DNA was extracted from tissues using the DNeasy Blood and Tissue kit (Qiagen). Due to sensitive political and conservation issues, we are unable to provide exact geographic coordinates corresponding to localities in Fig 1.

### DNA sequence data

We targeted two mitochondrial and four nuclear loci for DNA sequencing. PCR amplification of two mitochondrial loci (NADH-dehydrogenase 1, *ND1*; cytochrome-b, *cyt-b*) and two protein coding nuclear loci (prolactin receptor, *PRLR*; RNA fingerprint protein 35, *R35*) used previously published primers [36–38]. We used two additional anonymous nuclear loci designed from a genomic library enriched for microsatellite repeats: *scar298anl* (scar298anl.F: 5’-ATGGGAAGGCTTAAAATGAATC; scar298anl.R: 5’-TGTGACTTAGGGAACTGGGTATGT) and *scar875anl* (scar875anl.F 5’-CTTACCATTCAACCCTTCCTTG; scar875anl.R 5’- CTAGAGCAGACCAGTTCAATGTAAT). All PCR were conducted in 10 μl total volume. Annealing temperature for the new nuclear loci was 54°C.

We used 0.4 μl ExoSAP-IT (USB/Affymetrix) and 1.6 μl water to clean 5 μl of PCR product. One μl of clean PCR template was used in each cyclo-sequencing reaction using the same locus-specific primers used in amplification. Sequencing reactions were cleaned and run on an ABI 3730xl at the Duke Sequencing Facility. Chromatograms were verified and cleaned in Geneious R9 (https://www.geneious.com). Heterozygous sites in nuclear sequences were called with the appropriate ambiguity code. Sequences at each locus were aligned using the MAFFT [39] plug-in in Geneious. Sequences for unique alleles at each locus are accessioned on GenBank (MT795211 –MT795484).

Because the mitochondrion is inherited as a single unit without recombination, we concatenated the two loci (ND1 and Cyt-*b*) into a single alignment. Each of the four nuclear loci were treated independently. All sequences at each locus were aligned in Geneious and alleles at nuclear loci were determined using the program PHASE [40] and the helper program SeqPHASE [41].

### Microsatellite genotype data

Nuclear microsatellite loci were developed from a 454-library enriched for microsatellite motifs developed at Cornell University Evolutionary Genetics Core Facility. After initial screening of loci, we used the Qiagen Type-It microsatellite PCR kit to genotype individuals at these loci plus seven previously published loci [42] in five multiplex reactions (S1 Table). Forward primers for all loci were tagged with a fluorescent dye and samples were genotyped on an ABI3730xl at the Biotechnology Resource Center of Cornell University with GeneScan 500 LIZ size standard (Thermo Scientific). Alleles were called and verified for all individuals using GeneMarker 2.6. Prior to subsequent genetic analyses, all variable loci were tested for the presence of null alleles and selection by testing for Hardy-Weinberg Equilibrium (HWE) and for evidence of linkage disequilibrium using GenePop [43]. The final dataset included genotypes for all individuals at 27 variable and neutrally evolving nuclear microsatellite loci (Dryad, http://dx.doi.org/10.5061/dryad.sxksn0316)

#### Data analysis

*Summary statistics*. We used PAUP [44] to determine the number of parsimony informative sites for each sequence alignment and DNAsp v6 [45] to calculate the number of unique haplotypes, the number of segregating sites (S), nucleotide diversity (π), and the average number of nucleotide differences (k) for each sequence alignment.

*Haplotype networks*. We constructed parsimony networks in TCS [46] for complete mtDNA haplotypes for *S*. *arenicolus*. Because the results generated by network methods can be strongly influenced by missing data [47], we first omitted all individuals with missing sequence data for one of the two mitochondrial loci. We additionally omitted individuals for which we did not have locality information. The final haplotype network for mtDNA contained 195 individuals. We additionally constructed parsimony networks for the phased alleles at each nuclear locus.

*Phylogenetic analysis*. For the mitochondrial DNA, we estimated the phylogenetic relationships among *S*. *arenicolus* under both maximum likelihood and Bayesian frameworks. *Urosaurus ornatus*, *Uta stansburiana*, *Phrynosoma coronatum*, *Sceloporus jarrovii*, *S*. *merriami*, *S*. *occidentalis*, and nine individuals of *S*. *graciosus* were used as outgroups (following [5]). Concatenated mtDNA alignments were first reduced to unique sequences using an extension of the Python script *sequence_cleaner*.*py* [48] (modified script on Dryad, http://dx.doi.org/10.5061/dryad.sxksn0316). We estimated the best-fit model of sequence evolution at each codon position of each gene in DT-ModSel [49] and partitioned phylogenetic analyses by gene and codon position. The best fit models by DT-ModSel were a SYM+G for the first codon position of each gene, HKY+I for the second codon position of each gene, and TrN + I + G and TrN + G for the third codon position of *ND1* and *Cyt-b* respectively. We estimated the phylogeny under a Bayesian framework in MrBayes v3.2.6 [50,51] excluding individuals with missing data. TrN models were expanded to GTR for Bayesian analyses and the final analysis consisted of two independent runs each of 50 million generations sampled every 5,000 generations. All parameters were checked for adequate mixing and convergence, and the maximum clade credibility tree was summarized in MrBayes.

*Population genetic analysis*. We estimated within population diversity and among population pairwise F_ST_ for mtDNA as well as microsatellite data assuming membership to the phylogroups based on the Bayesian phylogeny. Estimates of F_ST_ were done in Arlequin [52] for mtDNA and in FSTAT for microsatellite data. Because samples were distributed evenly throughout the range of *S*. *arenicolus*, we additionally conducted population genetic analyses without any assumption of population membership using assignment methods in Structure 2.3.4 [53]. In Structure, we tested assignment of all individuals to *K* populations from K = 1 to 10. At each K we conducted 10 replicate runs each consisting of 1 million generations with the first 50% discarded as burn-in. We used StructureHarvester [54] to examine all runs and CLUMPP [55] and DISTRUCT [56] to visualize population membership. Structure runs with all individuals supported K = 2, so subsequent runs investigated further partitioning with each major group. For each subset of data, we tested K = 1 to 5 each with 10 replicate runs at each K each consisting of 2 million runs with the first 50% discarded as burn-in.

*Demographic analyses*. We estimated the historical demographics for each of five primary phylogeographic regions (A-E). These and finer-scale delineations of phylogeographic regions were based on geographic sampling and clustering of mtDNA haplotypes in network and phylogenetic analyses. We used multilocus sequence data to construct extended Bayesian skyline plots in BEAST 2.5.0 [57–59]. Each dataset included concatenated mtDNA alignments in addition to phased genotypes for each of the four nuclear loci. Substitution models for each locus were set based on MrModeltest [60] (S2 Table). All runs assumed a relaxed molecular clock with a log-normal distribution for the mtDNA partition and strict molecular clocks for the nuclear partitions. The rate for mtDNA was set with a log normal distribution with mean of 1x10^-8^ substitutions/site/year and SD of 0.27 [38]. Parameter trends were examined in Tracer to check for adequate mixing within runs and convergence across runs. Final runs were 50 million steps sampled every 5,000 steps for regions B, C, and E. The final runs for regions A and D were 100 and 200 million steps sampled every 10,000 and 20,000 steps, respectively. Extended Bayesian skyline plots we generated after discarding the first 25% sampled steps as burn-in.

*Hypothesis testing*. Based on the results of phylogenetic analyses and assignment tests, we tested three alternative hypotheses of divergence and population expansion among three geographic groups (Northern Mescalero Sands, Southern Mescalero Sands, and Monahans Sandhills) assuming that the Monahans Sandhills populations were ancestral and of constant population size [35] (S1 Fig). We used approximate Bayesian computation to evaluate support for these models and estimate demographic parameters of the best supported model in DIYABC [61]. Analyses included mtDNA and phased nuclear sequences. Locus parameters were specified after estimation of substitution models for each locus in DT-ModSel. The prior for the mtDNA mutation rate was set as a normal distribution with a mean of 1x10^-8^ substitutions per site per year [38] and nuclear substitution rates were set as uniform distributions. Initial runs were used to determine adequate priors for demographic parameters. The final analysis included 2 million samples for each divergence model (6 million total) with a linear regression step to extract the closest 1% of samples and determine the best supported model of the three. For the best supported model, we used the same selection/rejection process to estimate divergence times and demographic parameters from the closest 1% of the 2 million samples.

## Results

### Summary statistics

Sample sizes, alignment lengths, the number of unique haplotypes, number of segregating sites, average nucleotide differences, and nucleotide diversity are reported in S3 Table. As expected, nuclear loci were less variable than mtDNA though nucleotide diversity was similar for mtDNA and two nuclear loci. We also recovered multilocus genotypes for 237 individuals at 27 variable microsatellite loci that conformed to HWE expectations and did not show any evidence of linkage or null alleles. The average number of alleles per locus was 16.4 with a range from 3 to 35 (S4 Table).

#### Haplotype networks and phylogenetics

**Fig 2:**
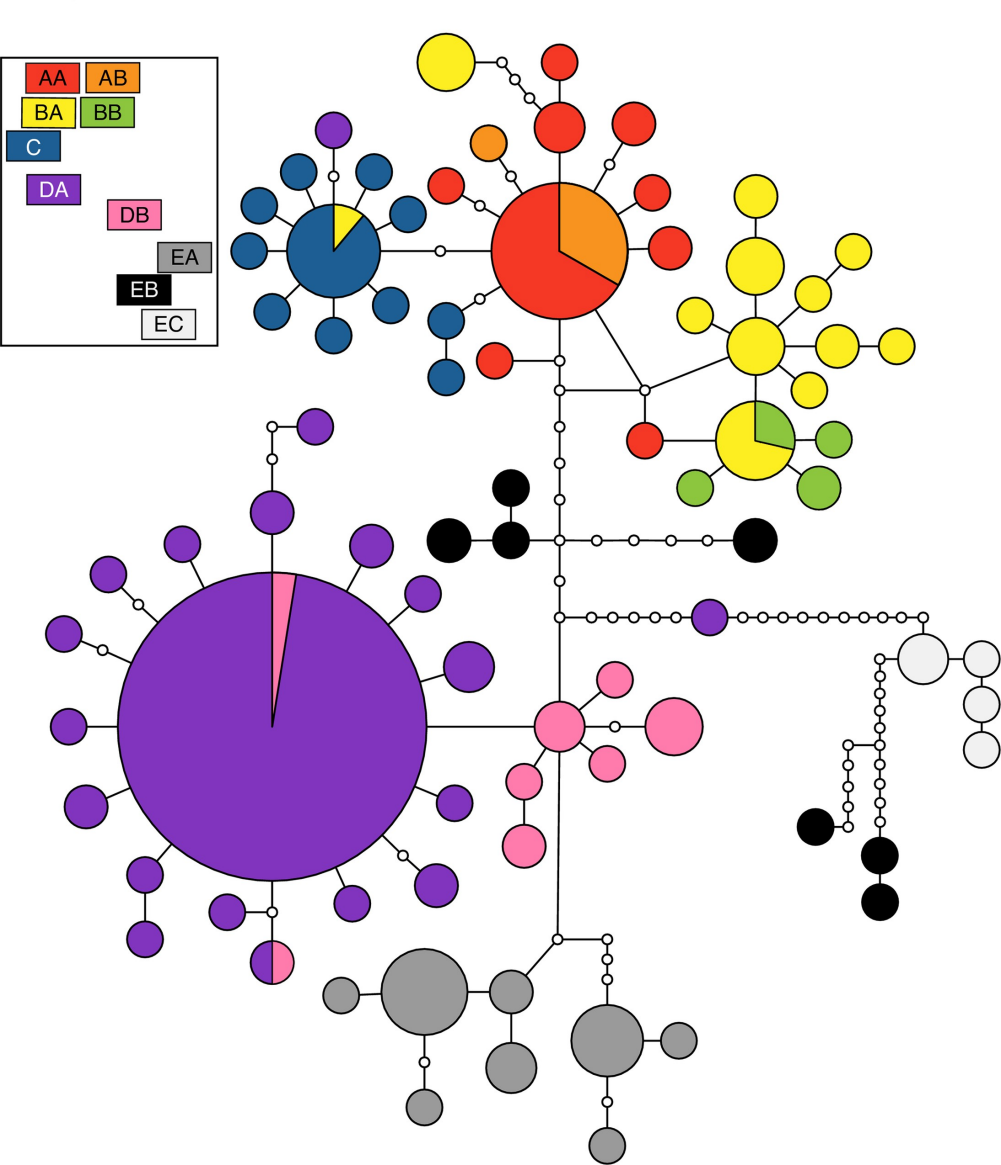
Haplotype networks based on concatenated mtDNA sequences. Circles represent unique haplotypes with the size of the circle corresponding to the relative abundance and the color referring to the region of origin of individuals with that haplotype (see boxes in upper left representing geographic approximations of each region). Lines connecting haplotypes represent one mutational step. Small white circles represent unsampled haplotypes. [Alternate version for those with color vision deficiencies; S2 Fig].

Mitochondrial haplotype networks revealed geographically associated haplotype groups for mtDNA that largely correspond to regions of grossly contiguous habitat (Fig 2; S2 Fig). In the Northern Mescalero Sands, there are three main haplotype groups corresponding largely with the A regions (Fig 1; AA and AB), the B regions (BA and BB), and the C region, though the genetic divergence among these three groups is small. Common haplotypes are shared across regions, but derived haplotypes are unique to each region. Regions AB and BB have genetic diversity that is primarily a subset of the diversity found in AA and BA, respectively. The Southern Mescalero Sands (Regions DA and DB) are genetically divergent from the Northern Mescalero Sands populations with the barrier between the two groups reflecting a west-east constriction in the distribution of potentially suitable habitat (referred hereafter as “the Skinny Zone”). Among the Southern Mescalero Sands individuals in region DA, we find a single widespread haplotype and multiple derived haplotypes. In addition, region DB at southernmost tip of the Southern Mescalero Sands contains a cluster of derived haplotypes.

Populations in the Monahans Sandhills are genetically distinct from all other *S*. *arenicolus* populations, but do not form a single haplotype group. There is high sequence divergence among haplotypes from the Monahans Sandhills despite occurring in a relatively restricted geographic area and they are distantly related to Mescalero Sands haplotypes. The EA and EC areas each have unique haplotypes without a single, most common haplotype. The EB haplotypes fall out into two main groups, one that is equally distant to northern and southern Mescalero Sands haplotypes and one that is distantly related to all other recovered haplotypes (Fig 2).

In general, nuclear gene regions had much lower genetic diversity with very little genetic structure (S3 and S4 Figs). Across all four nuclear loci, we found a similar pattern with the most common haplotypes occurring in most, or all regions. At PRLR and scar875, several derived loci were unique to Monahans Sandhills populations and Monahans Sandhills plus Southern Mescalero Sands populations. With one exception (AB locus R35) regions AA, AB, and BB did not have any unique nuclear alleles.

**Fig 3:**
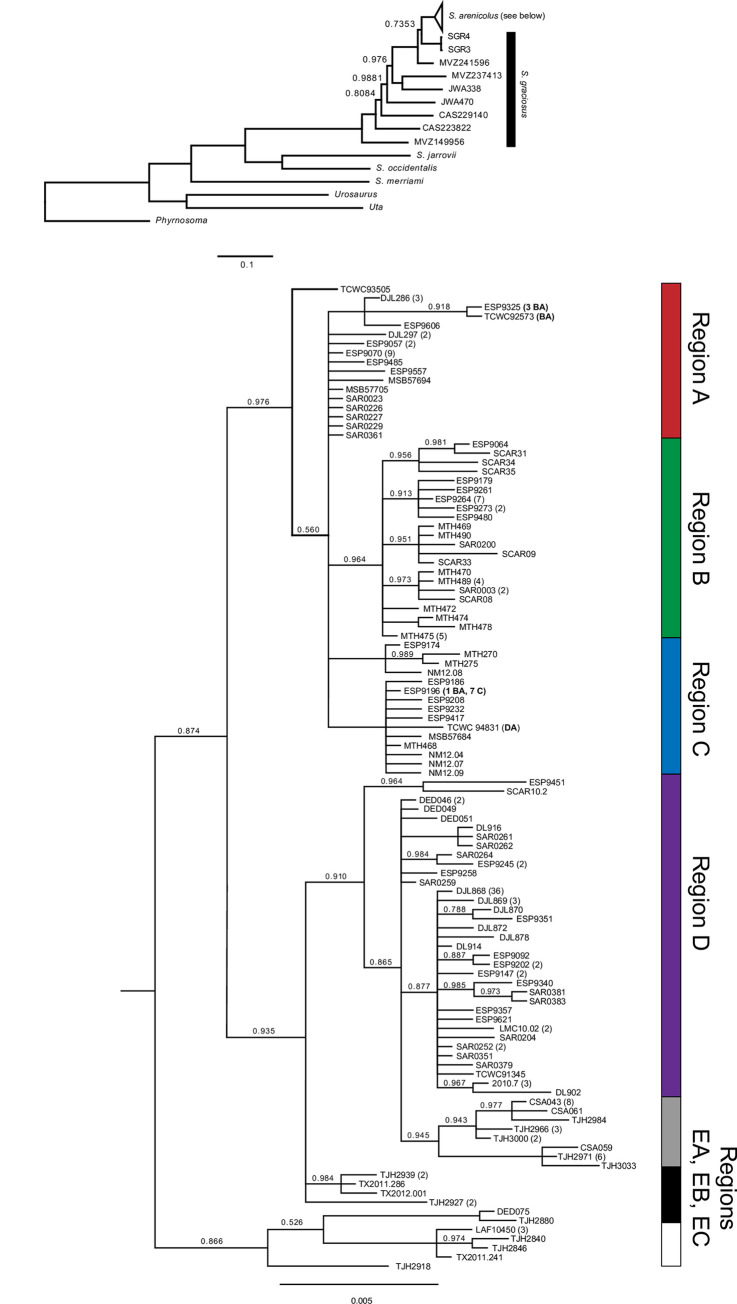
Majority-rules consensus tree from Bayesian phylogenetic analysis of concatenated mtDNA sequence data. Tips are labeled with a sample name followed by the number of samples with the identical haplotype. Regions of collection are indicated vertically with several exceptions listed parenthetically in the tip name. Posterior probability for all nodes is 1 unless otherwise indicated.

Phylogenetic reconstructions largely corroborated the groups found in the network analyses (Fig 3). We recover *S*. *arenicolus* as monophyletic (PP = 1). Monahans Sandhills populations were paraphyletic with respect to Mescalero Sands populations with the southern-most Monahans Sandhills individuals forming a weakly supported clade (PP = 0.866) sister to all other *S*. *arenicolus*. Among the remaining individuals, there is strong support for a Northern Mescalero Sands clade including individuals north of the skinny zone (PP = 0.976) and moderate support for a Southern Mescalero Sands–Monahans Sandhills clade that includes individuals south of the skinny zone and the northern and central Monahans Sandhills (PP = 0.935). Within the Northern Mescalero Sands clade, we recover support for some clusters of individuals, but do not find well-supported clades corresponding to distinct geographic regions. Individuals from region A, at the northern end of the range, form a basal polytomy relative to smaller, but otherwise well-supported clades containing most individuals from the remaining regions north of the skinny Zone and one individual at the northern end of region DA (Figs 1 and 3). Four individuals from the center of region BA are closely related to haplotypes from region A. Support for a clade that includes most B individuals is high (PP = 0.964) as is support for two different clades that each primarily include individuals from C (PP = 1). One of these two region C clades additionally had haplotypes of individuals from region BA (same haplotype as ESP9196) and DA (TCWC94831). We do not recover very much genetic resolution for individuals south of the skinny zone in the Southern Mescalero Sands or the northern or central Monahans Sandhills. There is not strong support (PP = 0.910) for a clade including individuals from regions D and EA. Notably, individuals from Monahans Sandhills are paraphyletic and their relationships largely unresolved. While most individuals from the Monahans Sandhills regions cluster with other individuals from the same region, as expected from the haplotype network, there are a few individuals that fall out with individuals from different regions. For instance, both DED075 and TJH2880 were collected from within the EB region, north and south of other EB individuals that form a clade. These two individuals fall outside the EB clade and potentially cluster with individuals from EC, though support for these clades is weak (Fig 3).

#### Demographic estimates

Extended Bayesian skyline plots match the inferences made from the haplotype networks (S5 Fig). We see evidence of recent population expansion in region D and demographic stability in region E. Regions A, B, and C show some evidence of population expansion though the credible intervals around the most recent population sizes is large and does not exclude the possibility of demographic stability.

#### Population genetics

Pairwise F_ST_ among populations was high among regions with values significantly different from zero ranging from 0.099 to 0.904 for mtDNA and 0.026 to 0.236 based on microsatellite loci (Table 1).

**Table 1:** Pairwise F_ST_ values among regions for microsatellite genotypes (above diagonal) and mitochondrial sequence data (below diagonal).

	AA	AB	BA	BB	C	DA	DB	EA	EB	EC
AA		**0.0807**	**0.0349**	*0*.*0689*	**0.0358**	**0.1443**	**0.1723**	**0.1488**	**0.1654**	**0.1457**
AB	0.0279		0.1077	*0*.*1911*	0.0749	**0.1988**	0.2374	0.2080	0.2356	0.2010
BA	**0.3389**	**0.3151**		*0*.*0512*	**0.0264**	**0.1337**	**0.1594**	**0.1349**	**0.1388**	**0.1033**
BB	**0.6593**	**0.7667**	**0.2147**		*0*.*0807*	*0*.*1643*	*0*.*2116*	*0*.*1688*	*0*.*1830*	*0*.*1592*
C	**0.4102**	**0.3688**	**0.4605**	**0.6583**		**0.1271**	**0.1523**	**0.1213**	**0.1350**	**0.1071**
DA	**0.8466**	**0.8584**	**0.8083**	**0.8687**	**0.8503**		**0.0411**	**0.0882**	**0.1188**	**0.0990**
DB	**0.8381**	**0.8395**	**0.7483**	**0.8439**	**0.8173**	**0.3311**		**0.1213**	**0.1554**	0.1302
EA	**0.7984**	**0.7723**	**0.7372**	**0.7842**	**0.7858**	**0.5767**	**0.4206**		**0.1188**	**0.1017**
EB	**0.5554**	**0.4000**	**0.5021**	**0.4513**	**0.5523**	**0.6668**	**0.4663**	**0.5302**		0.0701
EC	**0.8406**	**0.8125**	**0.7540**	**0.8216**	**0.8099**	**0.8872**	**0.8424**	**0.8108**	**0.4119**	

Values significantly different from zero (at alpha < 0.05) are indicated in bold. The significance of some F_ST_ values could not be determined because of low genetic variability in region BB, indicated with italics.

**Fig 4:**
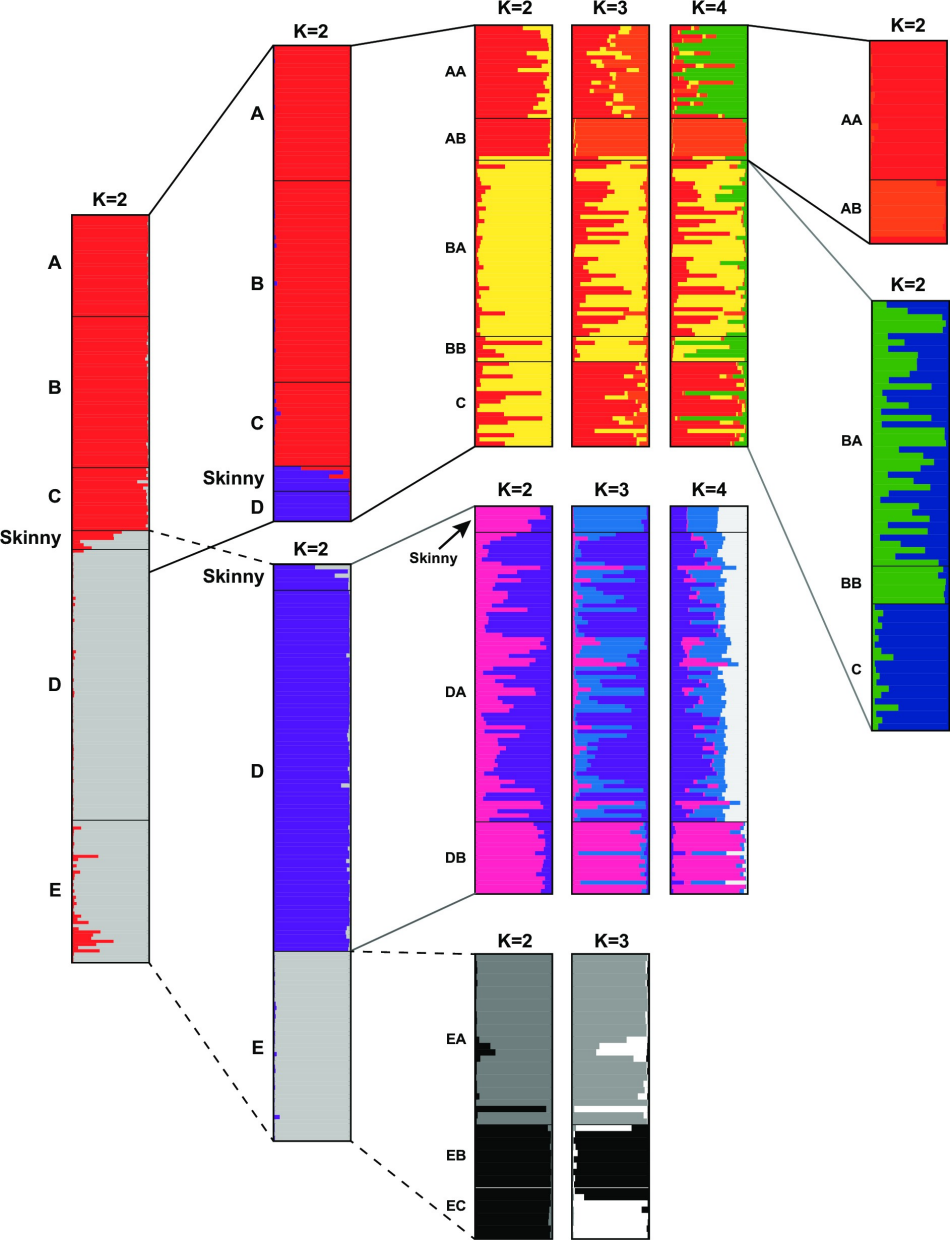
Individual assignment plots from nested Bayesian assignment tests in STRUCTURE. Results at alternate values of *K* are shown for some subsets of individuals.

Assignment tests based on microsatellite data reveal nested structure at multiple spatial scales (Fig 4). Across all samples, our analyses recover two groups with some admixture. The geographic break between these two groups corresponded to the Skinny Zone of the Mescalero Sands with some individuals in this area being admixed. Further assignment tests in Structure with nested subsets of the data indicate that these admixed individuals are aligned with individuals in the Southern Mescalero Sands. We recover distinctive groups in the Northern Mescalero Sands with some admixture as well. Region A individuals are distinct from region B + C individuals although, assignment plots suggest some admixture between western A populations (AA) and populations in region C, corroborating results from the mtDNA haplotype networks. Analysis of the AA-AB groups recover AB as genetically distinct corroborating F_ST_ estimates (Table 1). Analysis of the BA-BB-C group supports BB and C as distinct from one another with BA having genetic affinities to both. Together these results show a clear genetic break between the Southern Mescalero Sands and Monahans Sandhills populations. For the Southern Mescalero Sands populations there is an additional genetic break between regions DA and DB coinciding with another constriction in suitable habitat. Among Monahans Sandhills samples, EA individuals are distinct from EB+EC. Individuals from EB and EC are somewhat distinct from one another though not all individuals within a region cluster unambiguously with others in the group.

#### Hypothesis testing

**Fig 5:**
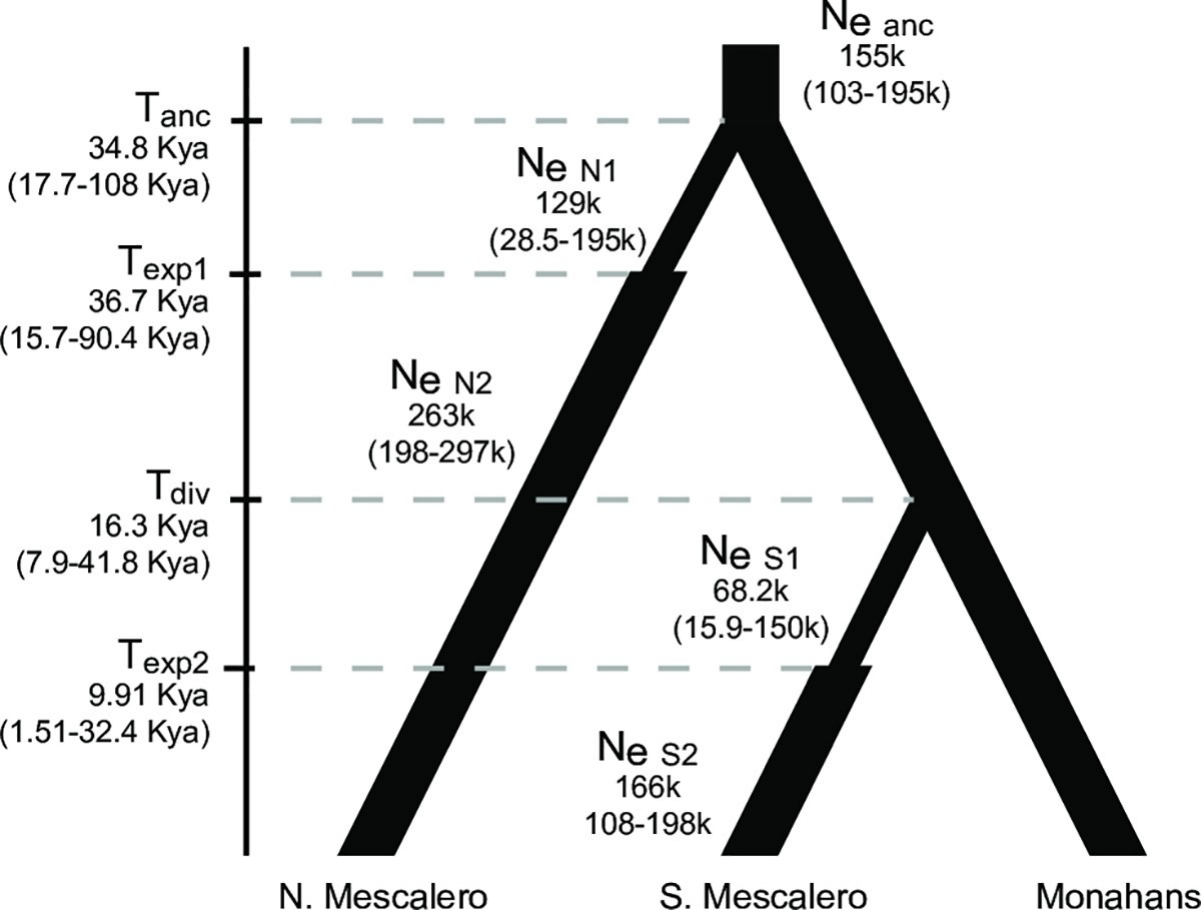
Demographic estimates for best supported model in ABC analyses. Scenario best supported by ABC analyses. Estimates of divergence and expansion times as well as current and historical effective population sizes are indicated.

We recover strongest support (PP = 0.9991) for a divergence scenario that involved colonization of the Northern Mescalero Sands from Monahans Sandhills populations around 34.8 Kya (CI 17.7–108 Kya) followed by colonization of the Southern Mescalero Sands from Monahans Sandhills populations more recently, around 16.3 Kya (CI 7.9–41 Kya; Fig 5). It is possible that the initial colonization of Northern Mescalero Sands included colonization of the Southern Mescalero Sands, with subsequent local extinction and recolonization, or genetic replacement. It is important to note that the 95% credible intervals for all estimates of divergence time and population size are broad. In fact, though the time of expansion in the Northern Mescalero Sands (T_exp1_) was constrained in individual ABC simulations to occur after the divergence between the Northern Mescalero Sands and the other regions (T_anc_), the median estimate for the former T_exp1_ precedes the median divergence time, T_anc_ (Fig 5), though both estimates have extremely broad and overlapping credible intervals.

## Discussion

Sampling of *S*. *arenicolus* throughout the entire range provides greater resolution of the evolutionary patterns of divergence of this narrowly distributed habitat specialist. We find support for multiple genetic groups within *S*. *arenicolus* suggesting limited migration in this habitat specialist. In particular, we find genetic structure beyond the three mitochondrial groups described in Chan et al. [35]. Patterns of divergence recovered by mtDNA corroborate nuclear microsatellite data and demonstrate the importance of the landscape-scale configuration of areas of habitat on the phylogeographic structure of this habitat specialist. With thorough geographic sampling we are able to identify regions that have served as barriers to population connectivity and characterize historical demographics across evolutionary time scales.

Lineages of *S*. *arenicolus* in the Mescalero Sands and Monahans Sandhills have independent and distinct histories that are associated with the timing of sand deposition and dune formation in these sub-regions. Indeed, the Mescalero Sands and Monahans Sandhills have related, but distinguishable geologic histories [62–64]. Both Mescalero Sands and Monahans Sandhills are sand sheets of the Southern High Plains deposited over older, compact eolian deposits comprising the Black Water Draw formation (204–43 Kya; [63]. Sand accumulation and dune formation has occurred repeatedly with current sand sheet age estimates for Mescalero and Monahans as 29.2 and 22.2 Kya respectively and with a more recent deposition ~ 7.5 Kya [63]. While estimated divergence times rely on the substitution rate that we assumed, they nonetheless help elucidate the historical demographics of *S*. *arenicolus*. Though *S*. *arenicolus* sampled from the Monahans Sandhills do not form a monophyletic group, it is clear that they are distinct from Mescalero Sands populations with estimated initial divergence between these two regions occurring long ago (34.8 Kya, CI 108–17.7 Kya; Fig 5). While the estimated divergence is older than the estimated age of the most recent sand deposition, this is a dynamic landscape that has undergone cycles of sand deposition during periods of aridity [63,65] such that this divergence is most likely associated with previous episodes of sedimentation and dune formation. There are broad CI around estimates of divergence and population expansion, but these estimates generally coincide with the sand age of Northern Mescalero Sands. Furthermore, the estimate of the most recent deposition falls within the CI for colonization and expansion times for the Southern Mescalero Sands.

The location and movement of sand dune formations has changed over millennia [62,64,66]. While the presence of sand dunes alone does not indicate the presence of shinnery oak-sand dune ecosystem, the distribution of habitat suitable to *S*. *arenicolus* has likely shifted in its occurrence and connectivity over geologic time. The current range map (Fig 1) is informed by currently occupied habitat, but potentially suitable habitat connecting regions may have occurred in the past. Given the dynamic nature of the landscape to which *S*. *arenicolus* is endemic, it stands to reason that dynamic histories also characterize the phylogeographic and population genetic structure in this species. Though the age of the Mescalero Sands and Monahans Sandhills geologic formations are uncertain, our data suggest that Monahans Sandhills was the source population from which Mescalero Sands *S*. *arenicolus* populations were colonized. Mescalero Sands populations, which lie to the north of the Monahans Sandhills, are comprised of at least two distinct lineages, but nuclear microsatellite data and ABC analyses suggest that the Southern Mescalero Sands populations are more closely related to the Monahans Sandhills populations than to northern Mescalero Sands populations. The two sand formations are not currently connected by suitable habitat (Fig 1), but were presumably connected in the past facilitating the colonization of Mescalero Sands from Monahans Sandhills by *S*. *arenicolus*.

Our genetic data suggest that the colonization event associated with the current Southern Mescalero Sands populations occurred separately from the event that resulted in the Northern Mescalero Sands populations. Colonization of the Northern Mescalero Sands and divergence from the Monahans Sandhill source population is estimated to have occurred approximately 34 Kya followed by population expansion (Fig 5; S5 Fig). While recognizing that there are broad confidence intervals around the estimated time of this event, it is plausible that this divergence was associated with the deposition of loose aeolian sands over the Blackwater Draw Formation [63]. The second divergence was between the southern Mescalero Sands and Monahans Sandhills occurring later, around 16.3 Kya. This is similar to the age of more recent sand deposits in the Mescalero Sands, and subsequent population expansion with a median estimate of 9.9 Kya coincides roughly with the ages of the most recent aeolian deposits. This result suggests that after the colonization of the Mescalero Sands 34 Kya by *S*. *arenicolus*, habitat between the Mescalero Sands and Monahans Sandhills contracted or that Southern Mescalero Sands populations became extirpated and this area was later recolonized. Both scenarios seem plausible given what we know about *S*. *arenicolus* ecology and the dynamic nature of this system.

*Sceloporus arenicolus* requires interconnected shinnery-oak blowouts to support populations [18,30]. Shinnery oak flats or isolated dune blowouts impede movements and isolate populations. The divergences that we see across the Mescalero Sands and Monahans Sandhills correspond largely with the geographic extent of potentially suitable habitat identified in several studies of *S*. *arenicolus* [22,23,31]. We are also able to reconstruct historical population demography and recover variable, and sometimes dynamic, histories across populations of *S*. *arenicolus*. For instance, we find support for a major genetic break that coincides with the Skinny Zone, a narrow constriction (~ 3 km wide) in the central Mescalero Sands. This narrow zone of habitat for *S*. *arenicolus* is indicative of a long-standing barrier to dispersal and is now a point of secondary contact between divergent Northern and Southern Mescalero Sands populations.

We find shallow divergence, but distinct genetic diversity among the Northern Mescalero Sands regions indicating that habitat suitability also impacts population genetic connectivity at these finer spatial scales. Populations in some of these regions, like AB and BB, have diverged in isolation, suggesting a founder effect in line with the major direction of sand dune movement [64]. We additionally confirm a recent colonization and subsequent rapid population expansion in the southern portion of the Mescalero Sands (Region D). Finally, among the Monahans Sandhills samples we documented highly divergent alleles, deep divergence among populations, and relative population stability. We recovered at least three divergent groups among the Monahans Sandhills individuals indicating limited movement among older populations retaining ancestral genetic diversity.

The historical demography and patterns of divergence are reflected in the microsatellite data as well as the more slowly evolving mtDNA sequence data indicating that population structure is the result of longstanding habitat dynamics and restrictions to gene flow at multiple spatial scales, not just more recent anthropogenic change. Demographic studies of *S*. *arenicolus* have emphasized the importance of a network of suitable habitat at multiple spatial scales to support metapopulation dynamics and population persistence [32]. Landscape-ecological analyses of presence and absence of lizard community membership across the Mescalero Sands demonstrated that landscape heterogeneity, not dispersal, explained community assembly and meta-community structure [25,26]. The occurrence of the habitat specialist *S*. *arenicolus* was a driver of this pattern. As such, because the fine-scale distribution of suitable habitat is critical for local presence of *S*. *arenicolus*, we suggest the composition and configuration of the landscape with respect to unsuitable habitat types determines patterns of genetic connectivity across the range. The divergences we detect reinforce that extensive habitat may be necessary to support gene flow among populations and that habitat quality and habitat configuration at finer scales may be of critical importance to identifying potential corridors. Importantly, it is clear that the shinnery oak-sand dune ecosystem is a dynamic landscape where the configuration of habitat patches can change over decades and millennia. We know from phylogeographic studies that specialists in changing environments undergo repeated episodes of isolation and divergence [11]. While it is impossible to reconstruct the specific, chronological habitat configuration for the Mescalero Sands and Monahans Sandhills, it is likely that networks of suitable habitat have diverged and coalesced repeatedly over time (e.g., [67]). Source-sink dynamics are important at local and contemporary spatial and temporal scales [30,33], and this may translate to evolutionary patterns of population genetic structure at broader spatial scales and longer time scales. Under this model, habitat patches shift in their extent and distribution over time due to geological processes. The divergence and coalescence of habitat patches across time results in repeated local extinction, population divergence, and recolonization. In support of this scenario, we find population genetic and demographic patterns that reflect such dynamic processes and their variability across the landscape. For instance, the Southern Mescalero Sands is a more rapidly shifting sand dune formation [62,64,66] in comparison to the sand sheets of the Monahans Sandhills formation which are more stable and less dynamic [68]. The Southern Mescalero Sands may be characterized by local extinction and recolonization whereas the slower movement of the Monahans Sandhills may maintain demographically stable and isolated populations over longer time periods.

The patterns of divergence and gene flow that we see in *S*. *arenicolus* are not surprising of a habitat specialist inhabiting a dynamic landscape. Based on demographic studies [18,33] and observations of *S*. *arenicolus* [31,69–71], individuals do not move large distances. Their strict habitat requirements, and the naturally patchy and temporally dynamic qualities of this habitat, suggests that populations should be subdivided. The nestedness of genetic structure in *S*. *arenicolus* mirrors the hierarchical nature of their habitat preference: individuals require suitable blowouts within a matrix of shinnery oak, and populations are supported by a network of connected shinnery oak-sand dune complexes. While the genetic consequences of metapopulation dynamics have typically been explored at fine spatial and temporal scales, our phylogeographic study shows that these metapopulation dynamics may also leave their signature at broader spatial scales, in this case, across the range of this endemic lizard.

## Conservation

The shinnery oak-sand dune habitats of Mescalero Sands and Monahans Sandhills have experienced severe habitat degradation and fragmentation, particularly in the southern portions of the range of *S*. *arenicolus* [18,19]. Recent ongoing fragmentation due to human activities (e.g. highways and caliche roads built for oil field development and sand mining) decreases connectivity among populations and interrupts metapopulation dynamics leading to extinction of local populations [18,19,30,32]. It is important to emphasize that the genetic data we present here reflect historical, not contemporary, processes and patterns of genetic connectivity. Recent and extensive ecological research (e.g. [18,19,25,30,32]) make clear that *S*. *arenicolus* requires contiguous shinnery oak-sand dune complexes for population persistence and that individuals do not disperse across areas that are not shinnery oak-sand dune. There is most certainly restricted gene flow and genetically independent populations within each phylogeographic regions that is not detectable in our dataset because of limitations to the temporal and spatial resolution of mitochondrial DNA sequences and microsatellite markers at fine-scales. Our findings highlight regions to be considered as genetically distinctive conservation units and underscore the unique genetic and demographic history of different regions within the range of *S*. *arenicolus*. Continued fragmentation of the shinnery oak-sand dune ecosystem increases the likelihood that ancestral diversity and unique evolutionary lineages will be lost.

## Supporting information

S1 Table Microsatellite locus information. Characteristics of microsatellite loci, primers used, and multiplex conditions for 31 loci originally screened. Annealing temperatures and including/exclusion of Q-solution refers to the Type-It Multiplex Kit (Qiagen). Twenty-seven of these loci were included in final analyses.
(PDF)

S2 Table Substitution models used in EBSP analyses. (PDF)

S3 Table Summary statistics for DNA sequence data. (PDF)

S4 Table Summary statistics for geographic regions based on microsatellite data. (PDF)

S1 Fig Alternative population divergence scenarios evaluated with ABC. (PDF)

S2 Fig Mitochondrial haplotype networks (alternate version). Alternative versions for individuals with color vision deficiencies. Circles represent unique haplotypes with the size of the circle corresponding to the relative abundance and the color/pattern referring to the region of origin of individuals with that haplotype (see boxes in upper left representing geographic approximations of each region). Lines connecting haplotypes represent one mutational step. Small white circles represent unsampled haplotypes.
(PDF)

S3 Fig Nuclear haplotype networks. Separate networks for each of the four nuclear genes sequenced. Circles represent unique alleles with the size of the circle corresponding to the relative abundance and the color referring to the region of origin of individuals with that haplotype. Lines connecting haplotypes represent one mutational step. Small white circles represent unsampled haplotypes.
(PDF)

S4 Fig Nuclear haplotype networks (alternate version). Alternate version of nuclear haplotype networks for individuals with color vision deficiencies. Separate networks for each of the four nuclear genes sequenced. Circles represent unique alleles with the size of the circle corresponding to the relative abundance and the color referring to the region of origin of individuals with that haplotype. Lines connecting haplotypes represent one mutational step. Small white circles represent unsampled haplotypes.
(PDF)

S5 Fig Extended Bayesian skyline plots. Analyses conducted in BEAST for each of the five major regions (A, B, C, D, and E) utilizing mitochondrial and nuclear sequence data.
(PDF)
