# Supplementary material for: Phylogeographic structure of the dunes sagebrush lizard, an endemic habitat specialist
Source: PLoS One. 2020 Sep 16;15(9):e0238194. doi: 10.1371/journal.pone.0238194 (PMC7494111; doi:10.1371/journal.pone.0238194)
Supplement: S1 Table — Characteristics of microsatellite loci, primers used, and multiplex conditions for 31 loci originally screened. Annealing temperatures and including/exclusion of Q-solution refers to the Type-It Multiplex Kit (Qiagen). Twenty-seven of these loci were included in final analyses. (PDF) [file pone.0238194.s001.pdf]

S1 Table. Microsatellite locus information.

Characteristics of microsatellite loci, primers used, and multiplex conditions for 31 loci originally screened. Annealing temperatures and including/exclusion of Q-solution refers to the Type-It Multiplex Kit (Qiagen). Twenty-seven of these loci were included in final analyses.

Multiplex	Locus Name	Dye	Repeat	Size	Primer Sequences (5'-3')	Notes
Multiplex 1<br>57°C annealing<br>Q solution	sarms0344	VIC	$(ACT)^{11}$	250	F: CTGAGGACTTTGGTTTGAGGAA<br>R: ACCCAGTATGAGAGGAATGAAGC	
	sarms0473	PET	$(TG)^{13}$-T-$(TG)^{3}$	155	F: CCTCATCTGTATCCCTCTCATTAG<br>R: AGCCAGTCATCTTCTCTTTCATAC	
	sarms3490	NED	$(ATC)^{6}$	182	F: TTGCTTGTAACACCCACTGATAG<br>R: CCTGTACACCGCTATGATCAATG	
	sarms5185	6-FAM	$(GAT)^{5}$ GGT $(GAT)^{7}$	230	F: GCCAATGCAAGACAGAAAATAGAA<br>R: GGGAGGAGGGTTGGGATGC	
	sarms5839	NED	$(CTAT)^{15}$	335	F: ATGGCAGCTTTCTTTCTTG<br>R: TGTTATAGTCAGTGGGTTGGTA	
	sarms6346	PET	$(AGAT)^{11}$	369	F: ATGACTAATATCCACCCACCCTG<br>R: TTCAGCATGCTTGTAAGAACCTG	
Multiplex 2<br>60°C annealing<br>Q solution	sarms0506	6-FAM	$(TTG)^{6}$	206	F: TCAGGAAAAGGCGGGGTAT<br>R: GCAGGGAAAAACAGAGCAGAA	
	sarms2620	NED	$(AAGG)^{12}$	354	F: ACAACAGCATTTAGGAAGGAAGG<br>R: AAACTCCTACCTTCCTCGAACG	Failed HWE
	sarms2770	PET	$(AAC)^{8}(AC)^{3}$	190	F: TGGGTCACATACTCTCAGC<br>R: GCCATGCAGGAATACACT	
	sarms3645	6-FAM	$(GATA)^{18}$	408	F: ACTAGGTCTTCTTGTTCATCATTG<br>R: GGGCGGTACTCATCTCTGTTCCTA	
	sarms4015	PET	$(CTTT)^{12}$	421	F: CCCAGCCCAAAAGGAACC<br>R: CTCAGAGGCATCAAGAAGACA	
	sarms4354	NED	$(CA)^{18}$	160	F: TCCCTGATCACCCCTCTGC<br>R: TTGCTGAACGGTGATGTCTTG	
	sarms6064	VIC	$(AGT)^{7}$ ACT $(AGT)^{5}$	251	F: CAAGAACAGGGGGATGACTA<br>R: CAAGGGCAATGAACTCTGGA	
Multiplex 3<br>58°C annealing<br>no Q solution	sarms0739	PET	$(GT)^{10}$	163	F: GCAAAATGGAGAATCGTGTGAGTA<br>R: TAGTGGGGAATAGGAAGGGTAGG	
	sarms4884	6-FAM	$(TG)^{15}$	160	F: GTTTCCTTCATCTGTATCCTCTCA<br>R: AGCCAGTCATCTTCTCTTTCATAC	Failed HWE
	sarms5968	6-FAM	$(ACT)^{11}$	270	F: CAAGGGCAATGAACTCTGGA<br>R: GTGGTGGCGGCTCTGTG	
	sarms2196	VIC	$(AC)^{6}$	152	F: GATGATAGCTCCTTCAGTTGGTG<br>R: AGCAAATCATAGCACCACAGAAG	Monomorphic
	sarms7111	PET	$(TCTT)^{13}$	390	F: AACAAATGCCCCACC<br>R: GGCAAGACCCAAACACTA	

Multiplex 4<br>58°C annealing<br>no Q solution	sa60.12	PET	$(GT)^{24}$	122-148	From Chan et al. 2007	
	sarms0001	PET	$(GT)^{13}$	202	F: GGTTAGTTAGCATATTCCCATCC-3’<br>R: TATCATCACAGCAATTACTCCTTC	
	sarms0830	VIC	$(AC)^{8}$	166	F: AAGCAGAAATCAACACCTCTGTC<br>R: TAATGTGGCCTTGGATTCAGAAG	
	sarms3213	VIC	$(CTAC)^{2}$ CAT $(CTAC)^{14}$	404	F: CATTCTGGTCCCTGTGG<br>R: AATCATCTACCTCTTTACTG	
	sarms4545	NED	$(ATT)^{2}$ $(GTT)^{7}$	238	F: ACCACCACCATTCCCTTCTCCT<br>R: CATGCCTGGCCTTGCTTAG	
	sarms4547	6-FAM	$(AGAT)^{12}$	402	F: CATCTCTAGACTTGCTGCCAATC<br>R: AGGAGCAGCAATATACCTCTCAG	
	sarms5711	PET	$(AGAT)^{11}$	382	F: TTACTGAAATTCCTCTGGTCCAG<br>R: TACTGAAATCTCTTGTGTGCAGC	
	sarms0445	6-FAM	$(CT)^{10}$	145	F: AACAAGTCATGCCAGACTAAACC<br>R: AGAATATCATGGGGAACCTTGTC	Monomorphic
Multiplex 5<br>60°C annealing<br>no Q solution	sa52.20	VIC	$(TTG)^{9}$	103-112	From Chan et al. 2007	
	sa52.29	NED	$(TTTC)^{15}$	185-249	From Chan et al. 2007	
	sa60.02	NED	$(GT)^{10}$	117-133	From Chan et al. 2007	
	sar80	PET	$(ATAG)^{12}$ $(ACAG)^{6}$	349-402	From Chan et al. 2007	
	sar84	PET	$(CA)^{16}$	109-147	From Chan et al. 2007	
