# Supplementary material for: Phylogeographic structure of the dunes sagebrush lizard, an endemic habitat specialist
Source: PLoS One. 2020 Sep 16;15(9):e0238194. doi: 10.1371/journal.pone.0238194 (PMC7494111; doi:10.1371/journal.pone.0238194)
Supplement: S2 Table — (PDF) [file pone.0238194.s002.pdf]

S2 Table. Substitution models used in EBSP analyses

Phylogroup	mtDNA	PRLR	R35	scar298	scar875
A	HKY	F81	HKY+I	HKY+I	HKY
B	GTR	F81	HKY	HKY+I	HKY
C	GTR	F81	HKY	HKY+I	HKY
D	GTR+I	F81+I	HKY+I	GTR+I	F81+I
E	GTR+I	HKY	HKY+I	GTR+I	F81+I
