# Supplementary material for: Phylogeographic structure of the dunes sagebrush lizard, an endemic habitat specialist
Source: PLoS One. 2020 Sep 16;15(9):e0238194. doi: 10.1371/journal.pone.0238194 (PMC7494111; doi:10.1371/journal.pone.0238194)
Supplement: S3 Table — (PDF) [file pone.0238194.s003.pdf]

S3 Table. Summary statistics for DNA sequence data.

Locus	N	Alignment Length	Parsimony Informative Sites	Unique Seqs	Segregating sites, S	Average nucleotide differences, k	Nucleotide diversity, π
mtDNA	225	2097	72	78	106	8.72	0.0043
PRLR	208	541	9	12	9	0.95	0.0018
R35	195	658	5	12	8	0.71	0.0019
scar298	213	341	13	14	13	1.92	0.0057
scar875	212	287	12	20	13	1.27	0.0044
