# Supplementary material for: Phylogeographic structure of the dunes sagebrush lizard, an endemic habitat specialist
Source: PLoS One. 2020 Sep 16;15(9):e0238194. doi: 10.1371/journal.pone.0238194 (PMC7494111; doi:10.1371/journal.pone.0238194)
Supplement: S4 Table — (PDF) [file pone.0238194.s004.pdf]

S4 Table. Summary statistics for geographic regions based on microsatellite data.

Region	N	Expected Heterozygosity	Observed Heterozygosity	Average gene diversity	Mean number of alleles	G-W Index	Mean Allelic richness
AA	22	0.68755	0.63154	0.529	6.333	0.31496	2.393
AB	10	0.59348	0.51338	0.454	4.407	0.29101	2.171
BA	42	0.64902	0.57529	0.602	8.296	0.3371	2.560
BB	6	0.63409	0.41667	0.456	3.074	0.26077	2.103
C	20	0.65211	0.61647	0.647	7.593	0.31783	2.624
DA	76	0.6962	0.64657	0.684	10.815	0.37527	2.707
DB	16	0.64172	0.63093	0.593	6.667	0.3398	2.579
EA	27	0.67652	0.60591	0.649	7.852	0.34462	2.592
EB	10	0.66979	0.61111	0.670	5.667	0.33515	2.622
EC	8	0.73487	0.64263	0.653	5.667	0.32679	2.770
