# Supplementary figures and images for: Phylogeographic structure of the dunes sagebrush lizard, an endemic habitat specialist
Source: PLoS One. 2020 Sep 16;15(9):e0238194. doi: 10.1371/journal.pone.0238194 (PMC7494111; doi:10.1371/journal.pone.0238194)

# S1 Figure

**Scenario 1**

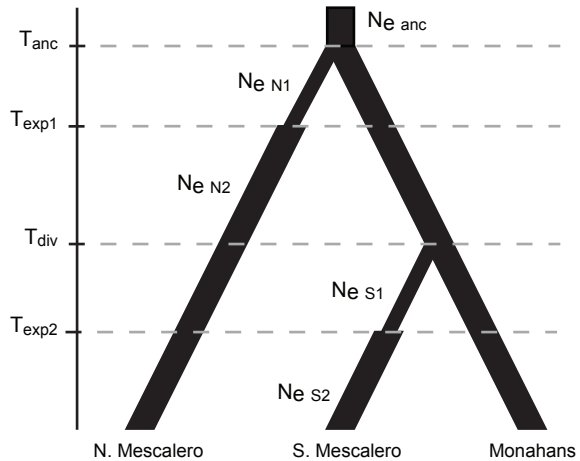

**Scenario 2**

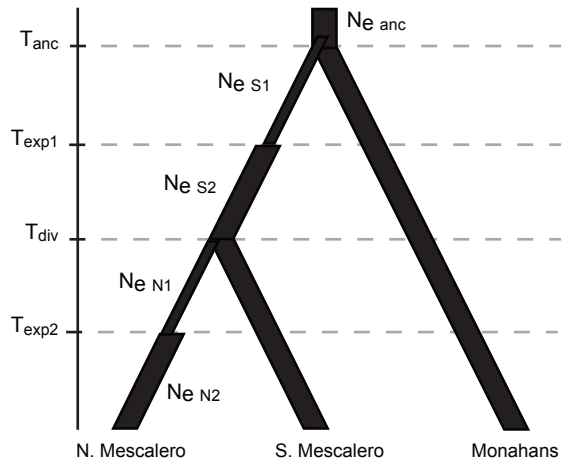

**Scenario 3**

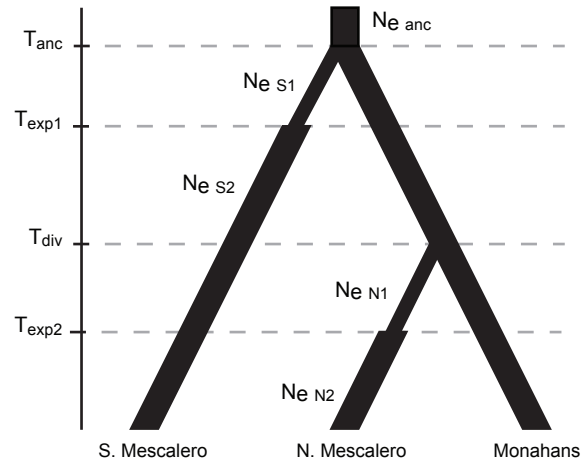

Supplement: S1 Fig — (PDF) [file pone.0238194.s005.pdf]

S2 Figure

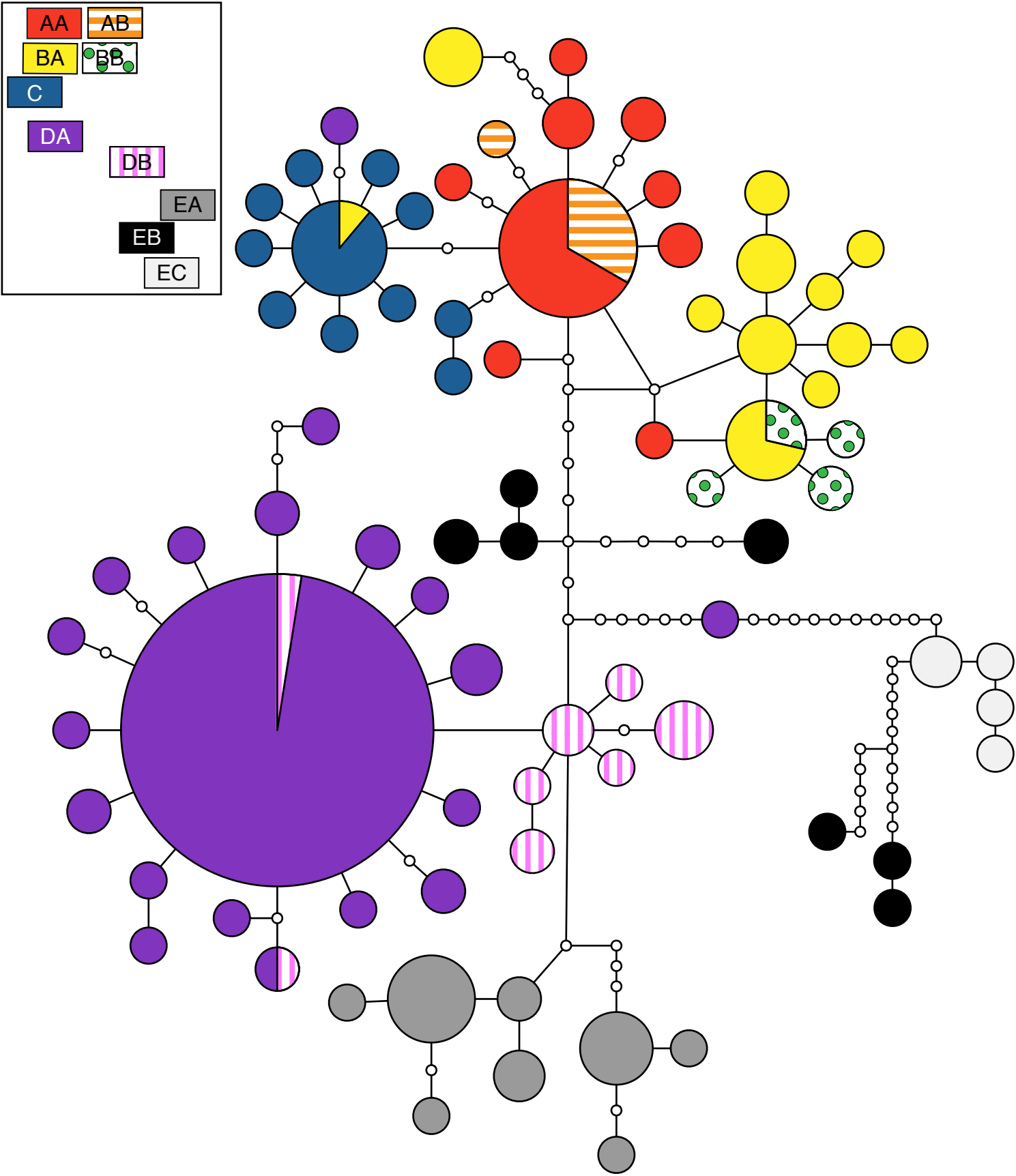

Supplement: S2 Fig — Alternative versions for individuals with color vision deficiencies. Circles represent unique haplotypes with the size of the circle corresponding to the relative abundance and the color/pattern referring to the region of origin of individuals with that haplotype (see boxes in upper left representing geographic approximations of each region). Lines connecting haplotypes represent one mutational step. Small white circles represent unsampled haplotypes. (PDF) [file pone.0238194.s006.pdf]

S3 Figure

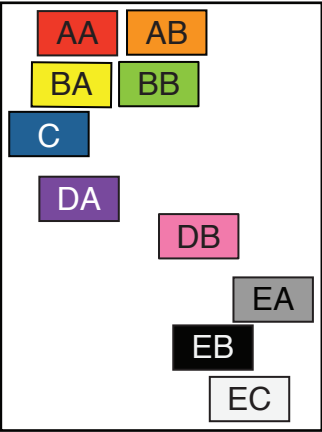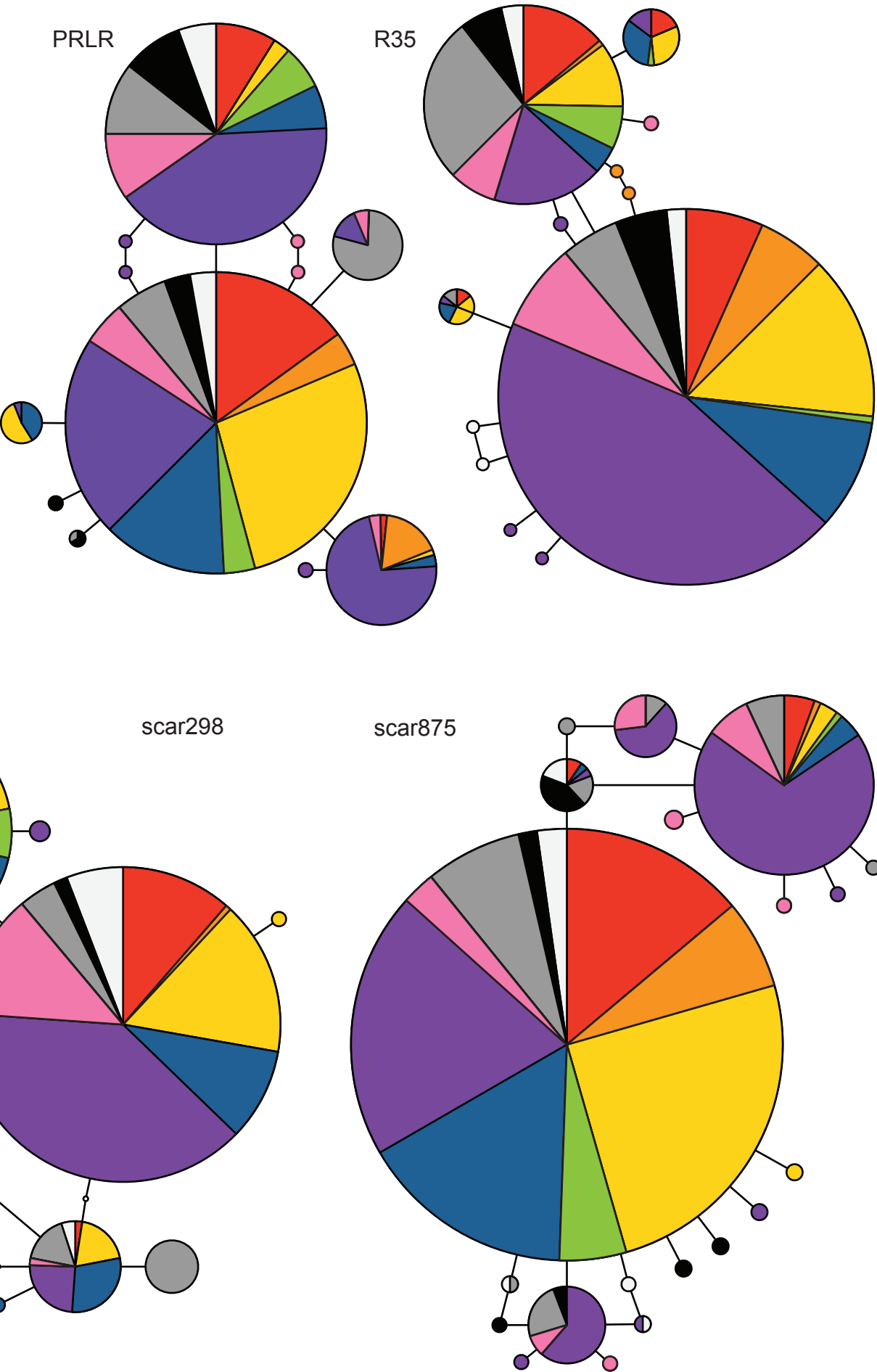

Supplement: S3 Fig — Separate networks for each of the four nuclear genes sequenced. Circles represent unique alleles with the size of the circle corresponding to the relative abundance and the color referring to the region of origin of individuals with that haplotype. Lines connecting haplotypes represent one mutational step. Small white circles represent unsampled haplotypes. (PDF) [file pone.0238194.s007.pdf]

S4 Figure

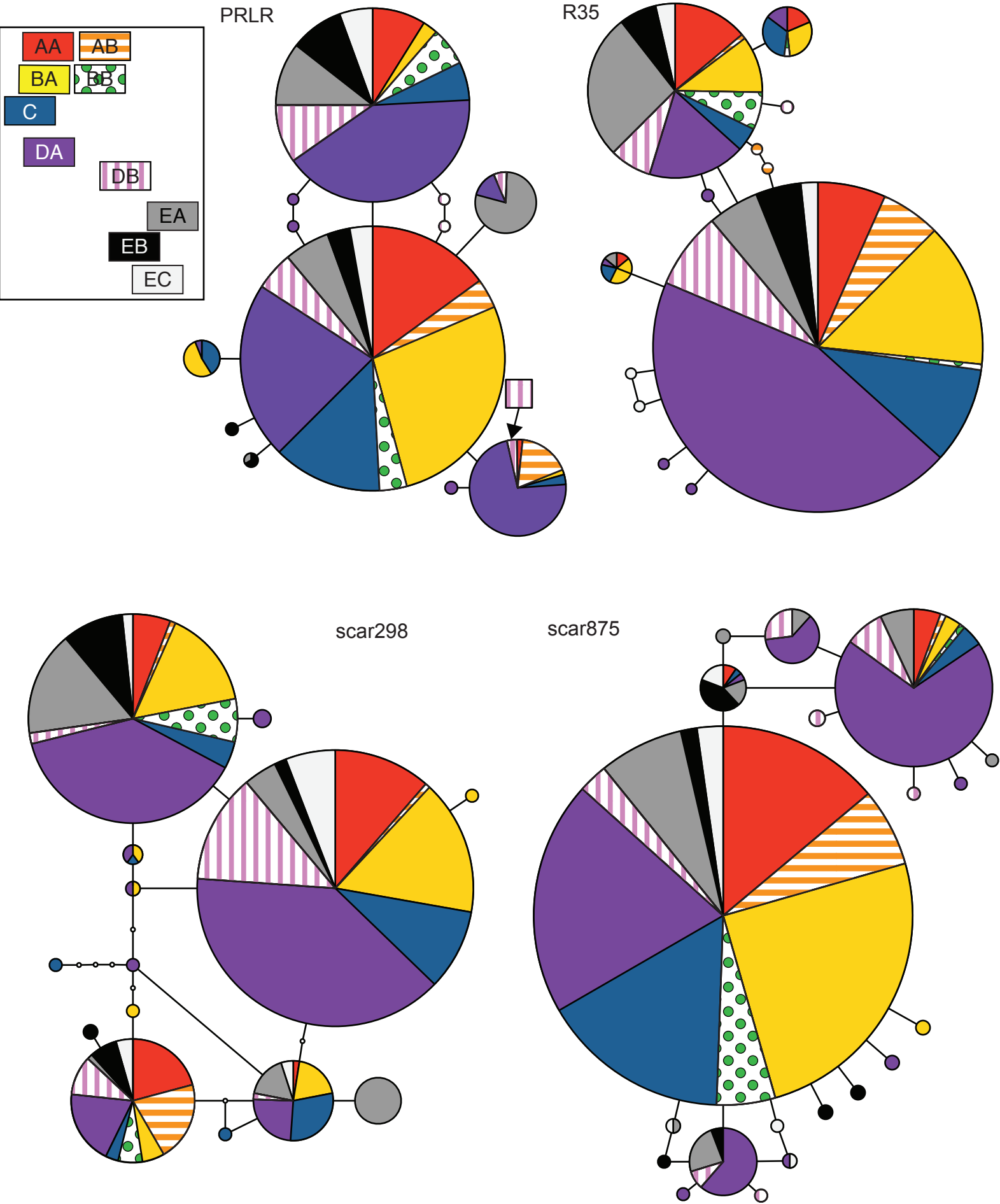

Supplement: S4 Fig — Alternate version of nuclear haplotype networks for individuals with color vision deficiencies. Separate networks for each of the four nuclear genes sequenced. Circles represent unique alleles with the size of the circle corresponding to the relative abundance and the color referring to the region of origin of individuals with that haplotype. Lines connecting haplotypes represent one mutational step. Small white circles represent unsampled haplotypes. (PDF) [file pone.0238194.s008.pdf]

S5 Figure

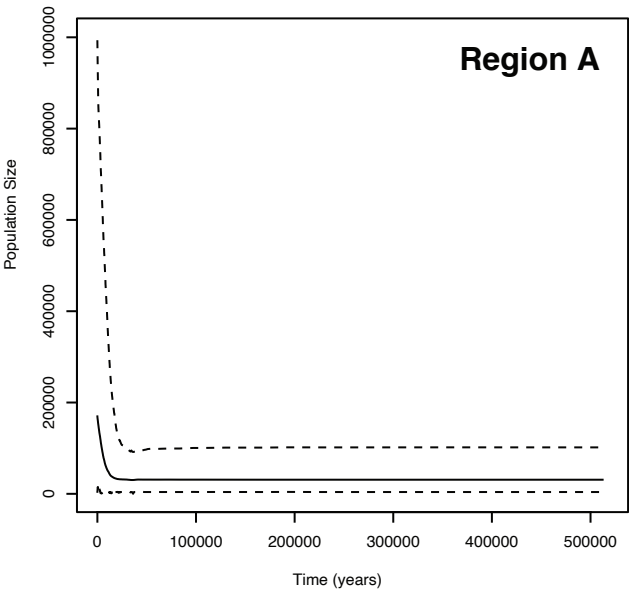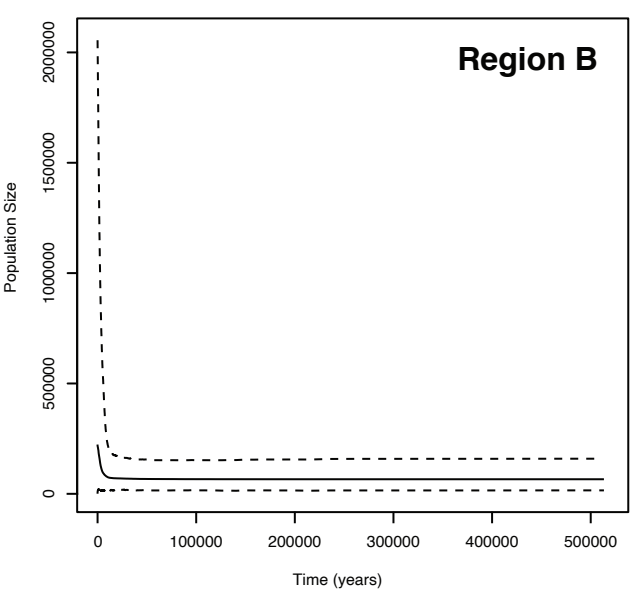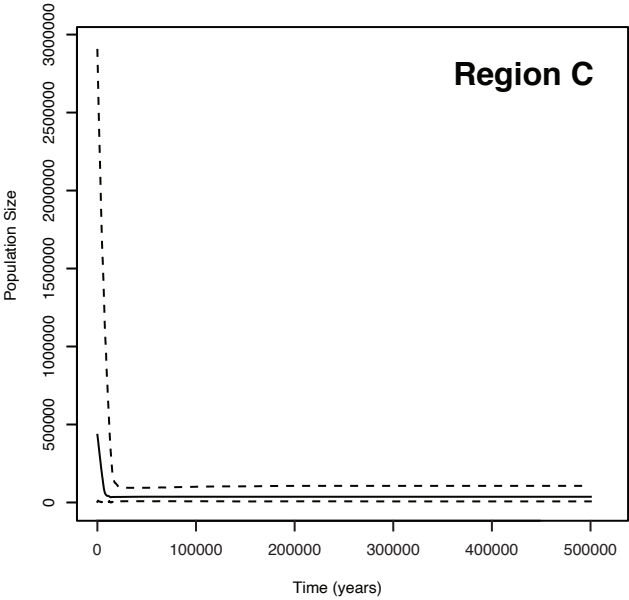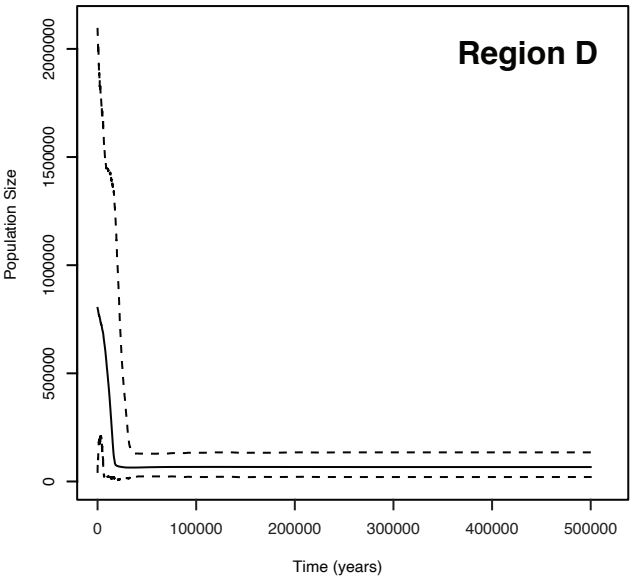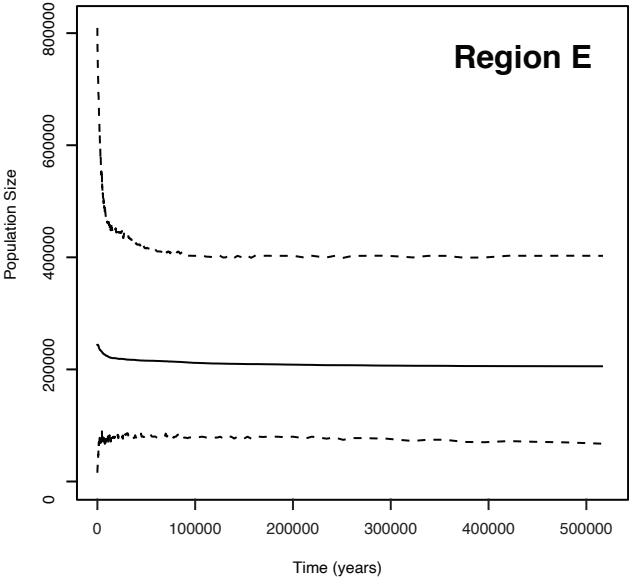

Supplement: S5 Fig — Analyses conducted in BEAST for each of the five major regions (A, B, C, D, and E) utilizing mitochondrial and nuclear sequence data. (PDF) [file pone.0238194.s009.pdf]
